# Time for a voluntary crisis research service

**DOI:** 10.1038/s41418-022-00968-3

**Published:** 2022-03-21

**Authors:** Joachim L. Schultze, Markus Gabriel, Pierluigi Nicotera

**Affiliations:** 1 Deutsches Zentrum für Neurodegenerative Erkrankungen (DZNE) e.V., Bonn, Germany; 2 PRECISE Platform for Single Cell Genomics and Epigenomics, DZNE and University of Bonn, Bonn, Germany; 3 International Center for Philosophy, University of Bonn, Bonn, Germany

The pandemic has mercilessly brought to light the inadequacies of how science is organized and prepared for fighting crises. Could a voluntary crisis research service (VCRS) be an answer?

On January 13^th^, 2022, 217845 articles were registered in PubMed [1–3] when searching for ‘COVID-19’ or ‘SARS-CoV-2’. On the same day, the world registered 317,166,137 positively tested individuals and 5,513,550 deaths [4]. It is time for new organization and preparedness of science for future 21^st^ century catastrophes. We provide some critical areas that require organizational adaptations and propose the VCRS.

**Fig. 1:**
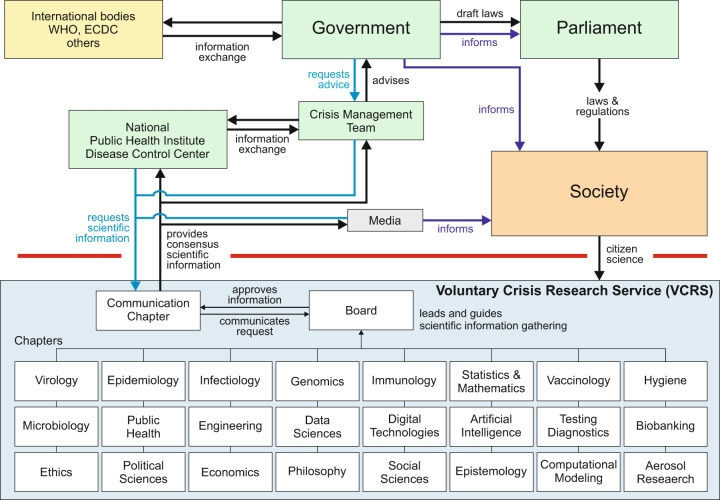
Outline of the Voluntary Crisis Research Service (VCRS) and its interaction with government bodies. The VRCS is divided into chapters, that are established in non-pandemic phases. Additional chapters can be developed and integrated. A board of the VCRS leads and guides scientific information gathering and prepares the information for the communication chapter, which is the only chapter communicating with the government bodies and the media. The VCRS could also foster citizen science during a pandemic. The consensus information from the VCRS is provided to national public health institutes or disease control centers, crisis management teams, if they are installed or government bodies including ministries themselves. A VCRS is not competing with freedom of speech of individual scientists, however, the scientific community via the VCRS would provide consensus information build by the expertise of hundreds of scientists provided in an interdisciplinary fashion.

Imagine firefighting services would have to write proposals to governments before reacting to wildfires. We cannot imagine such a scenario as we should not put ourselves at such a risk. Viruses killing millions must receive the same attention. Organizing networks of scientists can only be optimally achieved prior to a crisis. Funding of pandemic-adapted research needs to be quickly activatable following predefined plans and large enough research infrastructure needs to be recruited towards a transdisciplinary pandemic task force (Fig. 1). Countries that maintain core-funded research institutions such as the Helmholtz-Institutions in Germany covering many different disciplines in many research areas, or countries that have access to private funds (i.e. US, UK) are in advantage.

As much as we admire the enormous research on COVID-19, the scientific community followed its well-established competitive rules. Under non-pandemic conditions, we are in favor of scientific competition. We are less optimistic that it is the best solution when describing an emerging infectious disease with heterogeneous clinical courses. Instead, well organized large observational multi-center networks are the better strategy to get a complete picture. Such larger operations require strong team work which already needs to be established during non-pandemic times.

Teamwork needs practice. Consider a voluntary fire brigade would try to fight a wildfire without any prior practicing. Working as a network of individual brigades to fight large wildfires requires regular practice. To stay in the picture, in science we must establish and train teams before the crisis to only activate them at the beginning of the crisis.

It is possible to formulate the relevant questions in advance of a pandemic and to plan which experts are required to answer these questions. While most of us started science projects mainly on a local level, a preparedness plan would have accelerated efforts dramatically. Within the German COVID-19 OMICS initiative (DeCOI, decoi.eu), we quickly defined 8 projects in viral genome sequencing, functional genomics, and host genetics. However, we clearly underestimated the time and effort to obtain ethical approval. We are sure that colleagues around the globe faced similar challenges that slowed down progress.

Looking at early studies on COVID-19, validation principles were not sufficiently considered. Often single-center studies with insufficient numbers of patients were conducted to provide early access to new findings as a central element of crisis management. A more robust approach is activation of multi-center networks including validation cohorts. Following this approach in DeCOI we quickly obtained extremely robust findings even for highly complex analyses as single cell RNA-seq [5].

During the pandemic, societies learned that science is a continuous path of mutual correction of thesis and antithesis, which does not run in straight lines and has significant effects on society that cannot be anticipated and modeled by any single research area. Complex social systems counteract assumptions built into epidemiological models which by their very nature focus on properties of viral transmission at the neglect of socially organized behavioral modification in society. The world-wide discussion about masks is one example illustrating how knowledge gain lead to changes in recommendations [6]. The socio-political infrastructure is deeply interwoven with the public sphere of political debate, a crucial factor in the ongoing pandemic which must be integrated into scientific knowledge acquisition. While scientists are used to scientific dispute as part of scientific progress, society was overburdened by openly exhibited scientific disagreements. Looking at proven models of crisis communication, scientific communication needs improvement.

We propose the VCRS as a new model for science in context of crises built on principles established for other services fighting other crises, taking firefighting services as a paradigm. Many scientists in academia and in industry could be recruited to a VCRS providing their knowledge during a crisis. We postulate that a VCRS is more cost-effective then dedicated institutions, particularly considering the longer non-pandemic time intervals.

A VCRS is initiated nationally during the non-pandemic phase, embedded into international networks, and linked to organizations including the WHO, the world bank, the international monetary fund, and the United Nations. VCRS would be organized in chapters that are regularly evaluated for strengths and weaknesses and continuously adapted (Fig. 1). While AI specialists were not part of pandemic defense so far, AI has attracted attention during this pandemic as a major coping capacity [7–12]. Solutions such as Swarm Learning (SL) help utilizing world-wide data in a privacy-preserving fashion building trust without sharing data, while sharing insights thereby massively speeding up international collaboration [13].

A master plan describing the organizational structure, the members, the knowledge, expertise and technologies of the complete VCRS needs to be developed and continuously adapted to future threads with the ability to activate a crisis-adapted set of chapters during future crises (Fig. 1). Incentives, guidelines, basic requirements and rules including employer/employee relationships need to be established for scientists and institutions to become VCRS members.

During the non-pandemic phase, the VCRS board and the respective government agencies (e.g. CDC in the US or ECDC in Europe) continuously monitor e.g., spread of infectious diseases, and optimize and adapt VCRS plans. Countries could subdivide responsibilities concerning specific chapters to reduce cost. VCRS chapters have to simulate and prepare for crisis situations regularly, a scenario to be used to develop tools to diagnose potential pandemic viruses and build backbones for future vaccines thereby serving as innovation accelerators following scrum principles [14]. Evaluation results from such simulations are presented to the public.

In case of outbreaks, VCRS are alerted immediately to use the time efficiently until a pandemic might be declared. VCRS supports governments by providing scientific answers. Due to established chapter structures and regular practicing, VCRS research including surveillance programs, biosampling, data gathering, and clinical trials can start immediately.

Making scientific information public would also be different. We still suggest publication of research results following peer-review. Additionally, large data repositories would be generated to be utilized by other VCRS chapters for additional open research questions. Technical solutions such as SL preserving data privacy are activated to collaborate even faster [13]. Communication to governmental bodies, the public, the press and social media needs to be improved. Instead of individual researchers providing their own views, the VCRS would have a communication chapter handling any interactions with the press, the public, the governments, and social media (Fig. 1). Collectively, the VCRS is not intended to be confused with existing government-based structures executing regulations and laws. Rather the VCRS helps these government institutions with scientific data gathering, analysis and interpretation in a very structured fashion.

With the world population still increasing, future crises are foreseeable. Science and technological innovations are critical for coping with such crises. Consequently, the way we do science on the local, regional, national, and international level has to change.
